# A Generic Force Field for Protein Coarse-Grained Molecular Dynamics Simulation

**DOI:** 10.3390/ijms131114451

**Published:** 2012-11-08

**Authors:** Junfeng Gu, Fang Bai, Honglin Li, Xicheng Wang

**Affiliations:** 1 State Key Laboratory of Structural Analysis for Industrial Equipment, Department of Engineering Mechanics, Dalian University of Technology, Dalian 116023, China; E-Mail: jfgu@dlut.edu.cn; 2 Faculty of Chemical, Environmental and Biological Science and Technology, Dalian University of Technology, Dalian 116023, China; E-Mail: fangbai@yahoo.com.cn; 3 School of Pharmacy, East China University of Science and Technology, Shanghai 200237, China; E-Mail: hlli@ecust.edu.cn

**Keywords:** coarse-grained, force field, molecular dynamics, protein

## Abstract

Coarse-grained (CG) force fields have become promising tools for studies of protein behavior, but the balance of speed and accuracy is still a challenge in the research of protein coarse graining methodology. In this work, 20 CG beads have been designed based on the structures of amino acid residues, with which an amino acid can be represented by one or two beads, and a CG solvent model with five water molecules was adopted to ensure the consistence with the protein CG beads. The internal interactions in protein were classified according to the types of the interacting CG beads, and adequate potential functions were chosen and systematically parameterized to fit the energy distributions. The proposed CG force field has been tested on eight proteins, and each protein was simulated for 1000 ns. Even without any extra structure knowledge of the simulated proteins, the Cα root mean square deviations (RMSDs) with respect to their experimental structures are close to those of relatively short time all atom molecular dynamics simulations. However, our coarse grained force field will require further refinement to improve agreement with and persistence of native-like structures. In addition, the root mean square fluctuations (RMSFs) relative to the average structures derived from the simulations show that the conformational fluctuations of the proteins can be sampled.

## 1. Introduction

Over the last 30 years, the Molecular Dynamics (MD) method has played an increasing important role in dynamic behavior simulation of biomolecule at the atomic level [1]. In numerous application areas such as structural biology, biophysics, biochemistry, enzymology, molecular biology and medicinal chemistry, *etc.*, MD has become a major routine research tool. By means of MD simulation, biomolecular structure, kinetics, and thermodynamics can be investigated, for example, macromolecular stability, conformational and allosteric properties, the role of dynamics in enzyme activity, molecular recognition and the properties of complexes, ion and small molecule transport, protein association, protein folding, and protein hydration [2]. However, All-Atom Molecular Dynamics (AA-MD) is restricted severely by available computational capabilities because of the need of large amount of computing resources. In the 1970s, a small protein (bovine pancreatic trypsin inhibitor, composed of about 500 atoms) was first simulated, and lasted only about 10 picoseconds with AA-MD, limited by the computing power at that time [3]. With the development of modern computer technology, high performance computing and molecular dynamics method, the application of AA-MD has made great progresses in both space scale and time scale. Nowadays, AA-MD can simulate biomolecule system containing up to millions of atoms, with simulation time over microsecond level [4,5]. Despite this, AA-MD still cannot meet all the need of biomolecule research. Most dynamics and interactions within cells (e.g., protein-protein docking, rearrangement upon ligand binding, folding) occur on microsecond or even millisecond timescale, and usually involve large macromolecular aggregates. The simulation time of these processes is at least four to six orders of magnitude larger than the feasible time with AA-MD simulation, which has brought large barrier in the biomolecule simulation research.

In the past few years, Coarse-Grained Molecular Dynamics (CG-MD) methods for biomolecule have gained increasing attention [6–11]. The basic thought of CG-MD is to treat several or more atoms as a virtual particle (*i.e.*, so-called Coarse-Graining), so the huge quantity of degrees of freedom within complex biomolecule especially protein and the complexity of the corresponding force field will be decreased, therefore dramatically decreases the computational complexity of MD simulation. Various kinds of protein CG models and force fields have been introduced. Referenced to amino acid residue, protein CG models can be simply classified as multiple-point model [12–20], two-point model [21,22], one-point model [23–27] and much coarser multiple-residue model [28–30], and CG force fields varied from the simple harmonic potential to more realistic molecular force field. CG-MD has achieved plenty of research results, and has been applied in areas such as membrane [10,31,32], ion-channel [11,33], protein folding [34,35], and protein-protein interaction [36]. However, due to the limited speedup and reliability, the main available methods are difficult to be widely used in the simulation of large-scale biological systems to date, and the further development of CG-MD is still a challenge work for researchers. CG models need to be as simplified as possible in order to simulate more complicated biomolecules, while CG force field need to be as realistic as possible so that the kinetic behavior under AA-MD can be accurately reproduced. Current coarse graining methodologies are still not as predictive as AA-MD, because of the intrinsic difficulty in modeling the complex and diverse intra-molecular interactions with few parameters. Developing CG models and accurate force field for protein have become of great importance for studying large biological systems in both time and space scale.

As a representative CG force field, MARTINI has gained the most attention, and has been successfully applied to the simulations of protein and membrane systems. However, MARTINI still needs secondary structure restraints to maintain the stability of the native structure during the simulation, and the parameterization process of CG force field is too complicated and needs much experience, which usually needs quite considerable effort. Therefore, simpler and more efficient methods are continuously being researched. We report in our recent work on the improvement of CG-MD methodology. Novel CG models for protein simulation are designed, with which a residue is composed of only one or two beads, so the computational efficiency of MD can be improved significantly. A force filed based on the models is developed, based on known protein structures and AA-MD simulation results. Then the protein CG models and the force field are applied in MD simulations of eight small to medium size proteins. Finally, the simulation results are given and compared with those of AA-MD simulations and experimental values, indicating the effectiveness of the proposed CG models and force field.

## 2. Results

### 2.1. Results of Bonded Potential Parameterization

**Figure 1:**
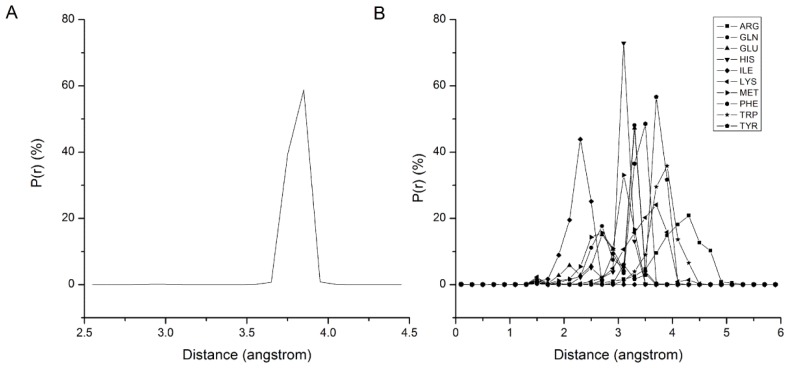
The bond length distribution of the *B*–*B* and *B*–*S_i_*. *B* denotes the backbone bead, and *S_i_* denotes the side-chain beads shown in distinct patterns.

In the bonded interactions, all the backbone beads are assumed to be the same, so the bond types can be classified as *B*–*B* and *B*–*S^i^*. The statistical results of bond length distribution are shown in Figure 1. Figure 1A shows that the *B*–*B* bond length is distributed in a narrow area from 3.6 Å to 3.9 Å and centered on 3.8 Å, so 3.8 Å is adopted as the equilibrium stretching length *L_bond_* in Equation 2 of *B*–*B*. Figure 1B shows the statistical results of distance distributions between 10 types of *S_i_* beads and their backbone beads. Each distribution shows a similar character with *B*–*B*, but the equilibrium length of *B*–*S_i_* bond is *S_i_* bead dependent. Table 1 summarizes the *L_bond_* of each *B*–*S_i_* bond adopted in our force field. The stretching energy profile of bond is extracted from the distribution of bond length with Boltzmann conversion method, and fitting with Equation 2 to get the force constant. The *B*–*B* force constant adopts an approximate value 100,000 kJ nm^−2^ mol^−1^, and the *B*–*S_i_* force constants adopt a mean value 5,000 kJ nm^−2^ mol^−1^ in our force field.

**Figure 2:**
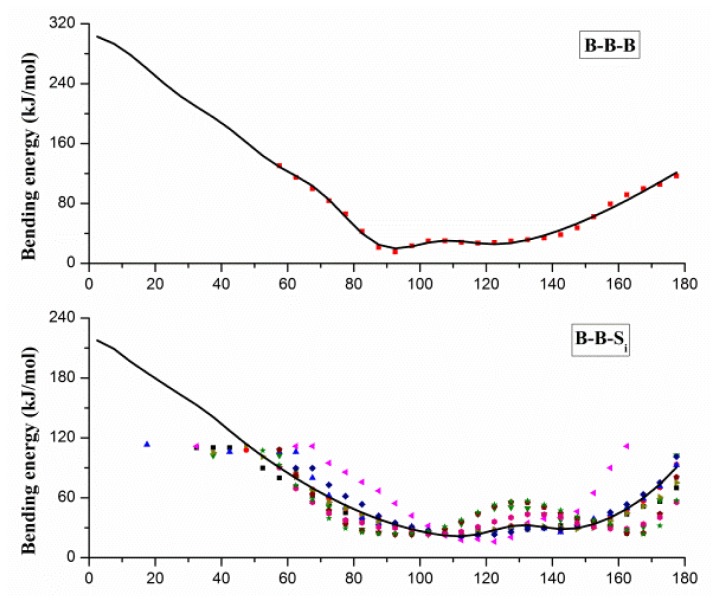

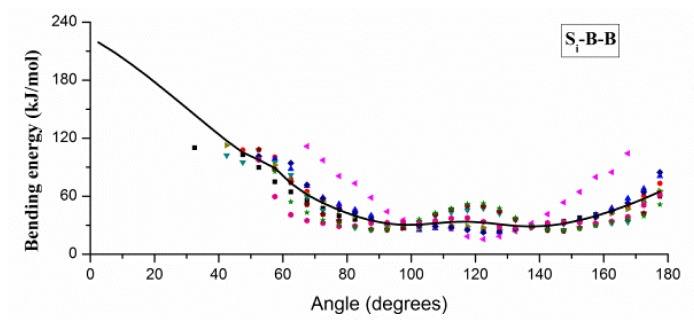
The angle bending energy profiles of *B*–*B*–*B*, *B*–*B*–*S_i_* and *S_i_*–*B*–*B* and the fitted potential function curves (black curves). *B* denotes the backbone bead, and *S_i_* denotes the side-chain beads shown in distinct patterns and colors.

The angles in CG protein system can be classified into three types: *B*–*B*–*B*, *B*–*B*–*S_i_* and *S_i_*–*B*–*B*, and angle bending energy profiles calculated from the probability distributions of these angels are shown in Figure 2, in which distinct colors and patterns are used to distinguish different *S_i_*. Two minima at about 90 and 120 degrees can be found in energy profile of *B*–*B*–*B* angle, which correspond to the α-helix and β-sheet secondary structure. A similar pattern of energy profiles is observed in Figure 2B,C, and only one set of parameters is used for *B*–*B*–*S_i_* (or *S_i_*–*B*–*B*) bending potential function. Due to the coarse-graining, we have to neglect some specific characters in the structure or energy distribution, and focus on the common characters behind the details. For fitting with Equation 3, the mean value smooth technique is adopted to handle different profiles in *B*–*B*–*S_i_* (or *S_i_*–*B*–*B*), and the fitted potential function curves are also shown with solid curves in Figure 2. Gaussian parameters in Equation 3 obtained from the fitting process are given in the Supplementary Materials.

**Figure 3:**
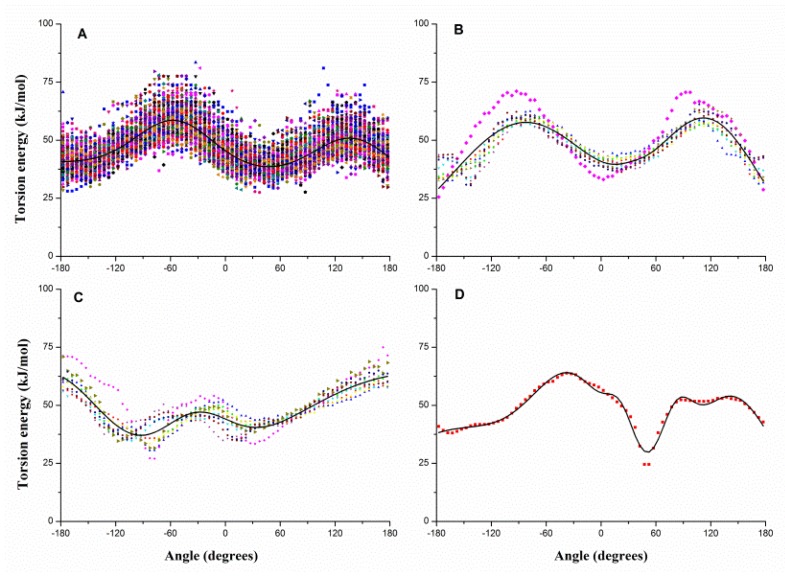
The dihedral torsion energy profiles of (**A**) *S_i_*–*B*–*B*–*S_j_*, (**B**) *S_i_*–*B*–*B*–*B*, (**C**) *B*–*B*–*B*–*S_i_* and (**D**) *B*–*B*–*B*–*B* and the fitted potential function curves (black curves). *B* denotes the backbone bead, and *S_i_*/*S_j_* denotes the side-chain beads shown in distinct patterns and colors.

Similarly, the dihedral can be classified into four types: *S_i_*–*B*–*B*–*S_j_*, *S_i_*–*B*–*B*–*B*, *B*–*B*–*B*–*S_i_* and *B*–*B*–*B*–*B*. Figure 3 gives the pseudo-dihedral torsion energy profiles of each type, e.g., Figure 3A shows the 100 energy profiles of *S_i_*–*B*–*B*–*S_j_*. Each type is fitted with Equation 3, and the fitting results are also shown with solid curves in Figure 3. Gaussian parameters for torsion potential are also given in the Supplementary Materials.

### 2.2. Results of Non-Bonded Potential Parameterization

**Figure 4:**
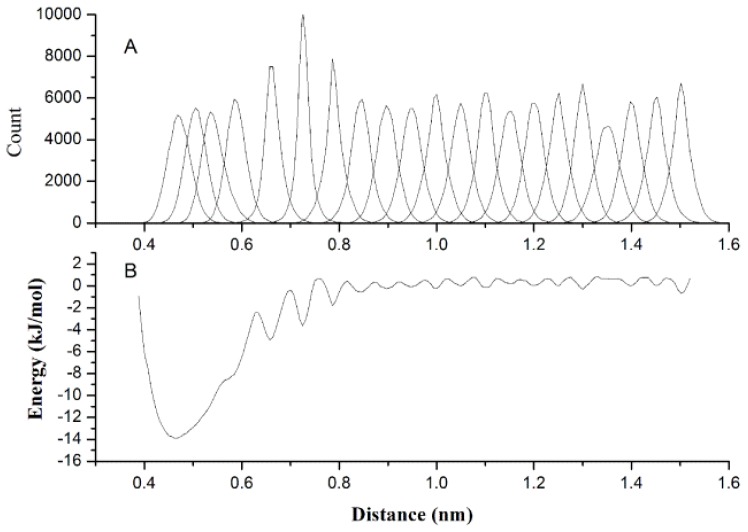
The histograms of the configurations within the umbrella sampling windows (**A**) and the potential of mean force against the distance of two ALA molecules (**B**).

**Figure 5:**
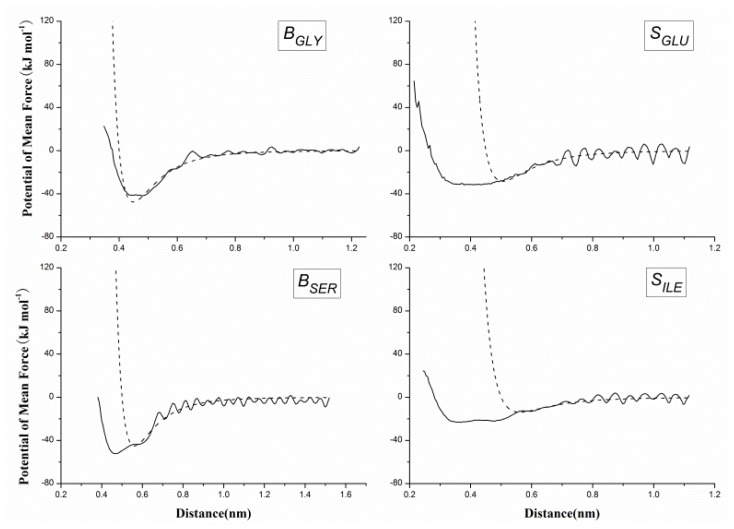
The potential of mean force between non-bonded homo pairs of coarse-grained (CG) beads (*B_GLY_*, *B_SER_*, *S_GLU_* and *S_ILE_*) against their distance, derived from umbrella sampling method with all-atom simulation (solid curves), and the van der Waals potential by fitting the potential of mean force with the Lennard-Jones function (dash curves).

It is important to accurately describe the non-bonded interactions of 20 CG beads in order to study protein folding and protein-protein interactions. US as a sampling improving technique was used to get the PMF between two homologue CG beads, and PMF curve is fitted to Equation 5 for extracting the best van der Waals interaction potential parameters. Figure 4A gives the histograms of the configurations within the umbrella sampling windows, which indicates there is sufficient overlap between adjacent windows. Figure 4B gives the PMF against the distance of geometric center of two *B_ALA_* beads, which have a minimum around 0.45 nm. However, when we made the statistical analyses of the distance distributions between two ALA amino acids on the above-mentioned protein structure database, the probability peak corresponding to the energy minimum was found around 0.55 nm. The reason for this inconsistency is that the CG bead is constrained by the surrounding beads while it is part of a protein, while is unrestricted in the US simulation. Most CG beads cannot be too close to each other in protein as in the US simulations, thus the short-range part of the PMF curve may not appropriate to model the non-bonded interactions in protein. However, the relatively long-distance interactions between CG beads are rarely affected by the environment in protein and can still be described by the PMF curves. Therefore, we made the statistical analyses of the distances for all 20 homologue CG bead pairs to determine the parameter *c_ij_* in Equation 5 when the van der Waals potential is equal to zero as listed in Table 2. Equation 5 was fitted to the PMF curve for determining the van der Waals well depth parameter with determined parameter *c_ij_*. Figure 5 gives the fitted results of CG beads *B_GLY_*, *B_SER_*, *S_GLU_* and *S_ILE_*. As in most cases, the position of the energy minimum determined by statistical *c_ij_* is farther than that of the corresponding PMF curve, the fitting is only noticeable in the long-range part of the PMF, as shown in Figure 5 (*B_SER_*, *S_GLU_* and *S_ILE_*).

### 2.3. Verification of the Force Field

To verify our force field, several proteins solvated in water were coarse-grained and simulated for a relatively long time. During the simulations, the maintenance of experimental structures and other thermodynamic properties are deemed to be indications of the feasibility of force field for protein simulation.

The test protein group is composed of eight small to medium size proteins which are not included in the protein set used for bonded potential parameterization. These proteins have recently been used to examine the performance of a modified version of the CHARMM force fields [37], and part of them have been used to test the PACE CG force field [14], so they were chose to verify our force filed convenient for comparison. All the CG-MD simulations were based on the GROMACS 4.0.5 package [38]. First, the protein was coarse-grained based on the proposed CG model, and topology files were generated with our developed scripts. Then the CG protein was solvated in CG water molecules, and the system was energy minimized with the proposed CG force field. Worthy of mention is that the GROMACS does not provide a Gaussian function type interface in its topology files, so user supplied tabulated functions were used for calculating the energy of angle bending and dihedral torsion. After the energy minimization, the CG system was equilibrated for 200 ps and then submitted for a 1000 ns simulation, using the canonical NPT ensemble at 300 K and 1 bar pressure, and the detail information for the eight simulated protein systems are listed in Table 3.

**Figure 6:**
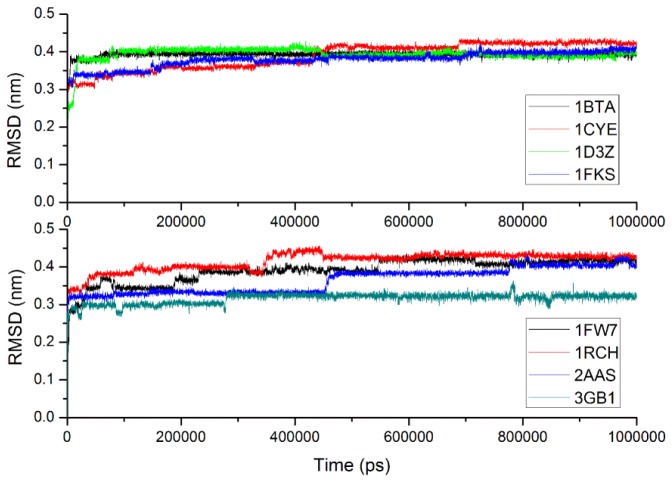
Resulting profiles of root mean square deviation of Cα carbons for eight proteins.

**Figure 7:**
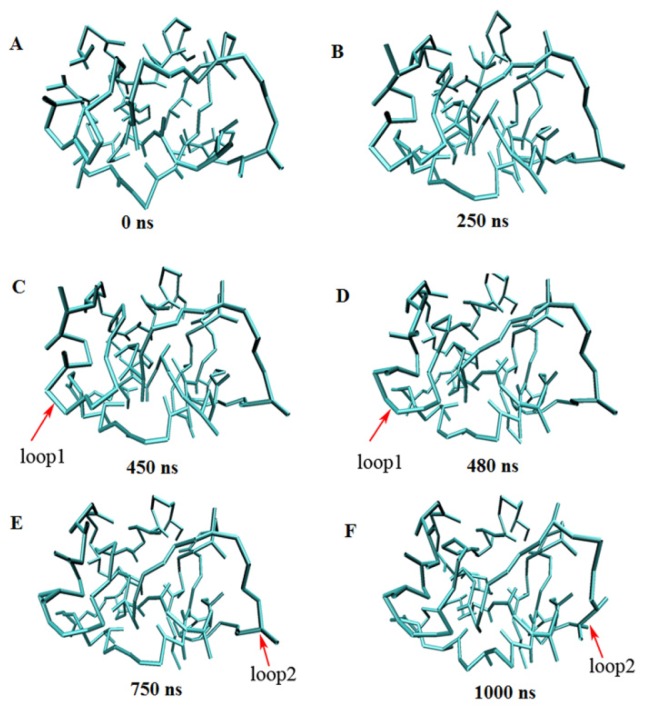
Snapshots of the 1000 ns coarse-grained molecular dynamics simulation for protein 2AAS at 0 ns (**A**), 250 ns (**B**), 450 ns (**C**), 480 ns (**D**), 750 ns (**E**) and 1000 ns (**F**).

Table 4 gives the simulation time and Cα RMSDs of eight proteins from their experimental structures derived from CG-MD simulations *versus* all-atom simulations. With the CG-MD methodology, eight proteins were all simulated for 1000 ns, and the average Cα RMSDs are varied from 0.316 to 0.415 nm, and the final RMSDs are between 0.323 and 0.431 nm. While with the all-atom simulation [37], eight proteins are simulated over 22–148 ns, and the average and final RMSDs is varied from 0.106 to 0.358 nm and 0.121 to 0.477 nm respectively. In general, the RMSDs with CG-MD are larger than those with AA-MD due to a longer simulation time and the roughness of our CG force field, but their values are comparable, and final RMSD of 1FKS is even lower. Thus, the experimental structures of proteins can be considered to be maintained with our CG force filed via long time MD simulations. Figure 6 gives the full trajectories of the Cα RMSDs of eight proteins. Most of the proteins reach their stable conformations within the first 100 ns and the Cα RMSDs are kept around 0.4 nm. In one case, the structure of 3GB1 is more stable and the Cα RMSDs are maintained around 0.32 nm, which mainly because few long loops are included in the native structure of the protein. It is noteworthy that the Cα RMSD trajectory of protein 2AAS has two distinct increases at around 460 and 800 ns. For analyzing the reason and investigating the conformation change during the simulation, the conformations of 2AAS at 0 ns, 250 ns, 450 ns, 480 ns, 750 ns and 1000 ns are sampled and plotted in Figure 7. From observation of the conformations at these 6 time points, skeleton structure of 2AAS is kept stable during the 1000 ns simulation, which also proves the native structure can be maintained with our CG force field. Comparing conformations at 450 ns and 480 ns, loop1 which is composed of residues 20–25 went through a large conformation change as indicated in Figure 7, which correspond to the distinct increase of Cα RMSD trajectory around 460 ns. The conformation change of residues 58–61 (labeled as loop2 in Figure 7) between 750 and 1000 ns corresponds to the RMSD value change around 800 ns. Both loop1 and loop2 are flexible loop regions located at the solvent-exposed surface of the protein, so they are less stable than the secondary structure and the hydrophobic core of the protein during the simulation.

**Figure 8:**
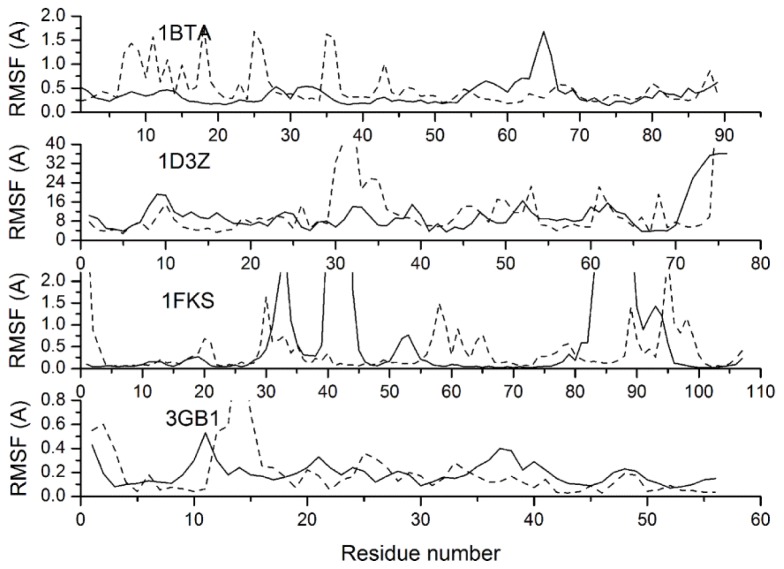
Resulting profiles of the residue root mean square fluctuations (dash curves) relative to averaged conformations compared with NMR experiments (solid curves) for proteins 1BTA, 1D3Z, 1FKS and 3GB1.

Another question that interested us is whether the conformational fluctuations of a protein can be reasonably simulated with our CG model and force field. In the PDB file of an NMR model, the B-factor column for each atom contains a measure how much that atom position varies throughout the models in the ensemble, which provides an experimentally detectable measure of equilibrium dynamics. Figure 8 gives the Root Mean Square Fluctuations (RMSFs) relative to the averaged structures for protein 1BTA, 1D3Z, 1FKS and 3GB1, which provide B-factors in their NMR structures. The RMSFs simulated with CG-MD are compared with B-factors via conversion equation RMSF^2^ = 3 × B/8/pi^2^, where B is the B-factor, which indicates the conformational stability degree. As shown in Figure 8, the RMSFs of protein 1D3Z and 3GB1 are consistent with the experimental values from a global perspective. However, at some locations of 1BTA and 1FKS, there are obvious inconsistencies between the simulated RMSFs and the experimental values: at residues 7–13, 15–20, 25–26 and 35–36 of protein 1BTA, the RMSFs are higher than the experimental values, while at residues 33–34, 40–44 and 84–91 of protein 1FKS, the situation is reversed. Through the analysis of protein structure and simulation trajectory, the above mentioned locations of 1BTA are either loops with lower curvature or ends of alpha helixes, while the locations of 1FKS are loops with higher curvature. The main reason of these conflicts is that the loop structure is mainly stabilized by the bonded interactions, while the bonded potentials adopted in our CG force field is a fitting of statistical average values due to the simplification. Therefore, loops with higher curvature are constrained by the bonded potential more strictly than they should be, while the situation of loops with lower curvature is opposite.

### 2.4. Efficiency of the Force Field

The main goal of coarse-graining is to improve the computational efficiency. For comparing the computational efficiency, the above mentioned eight testing proteins were simulated for another 10 ns with the proposed CG-MD methodology, AA-MD and MARTINI respectively. All the simulations were performed in serial on an Intel Xeon processor (2.4 GHz). The proteins were firstly centered in a box, the edge of which is 1 nm far from the molecules, and then solvated with water solvent. In all-atom simulations, GROMOS87 force filed and SPC water model were adopted, and a 2 fs time-step was used. In our and MARTINI CG-MD simulations, the corresponding CG water models and a 16 fs time-step were used. The energy of all the systems were minimized first, then equilibrated for 200 ps and submitted for a 10 ns simulation, using the canonical NPT ensemble at 300 K and 1 bar pressure. The simulation time is listed in Table 5. With each simulation method, the simulation time is proportional to the protein size (as listed in Table 3). The simulation time with our coarse-graining methodology is slightly less than that with MARTINI, which is mainly due to a coarser protein and water model. Compared with AA-MD simulation, MARTINI and our CG-MD method can achieve about 75~100 speedup. When more complicated solvent model is adopted in AA-MD, such as TIP3P, the speedup will be more obvious. It seems that larger time-step adopted in the CG-MD is a direct factor relating to the speedup, but the profound reason is that the appropriate coarse-graining model can maintain the structure stability of the protein in a CG-MD simulation with a larger time-step.

The average Cα RMSDs of all the simulations are also given in Table 5. The values of AA-MD here slightly differ with those provided in Table 4, which are got with a modified CHARMM force field and with a longer simulation period. With our simulations, the average RMSDs of AA-MD range from 0.128 to 0.259, which indicates AA-MD is most stable among these three simulation methodologies. The RMSDs with the proposed CG-MD methodology is lower than MARTINI for all eight proteins. Despite the fact that 10 ns is a relatively short simulation period, the results show that the proposed CG-MD methodology a comparable even better ability of native structure maintenance compared with the popular MARTINI CG force field. It should be noticed, the information of secondary structure is required in the simulations with MARTINI, while not required with the proposed method. It indicates that interactions in the CG protein structure can be balanced even without any extra structure restraints, and this makes the proposed model more suitable for simulating random or extended structures.

In addition, for evaluating the role of the CG solvent in the simulations, the testing proteins were also simulated in vacuum, and the average Cα RMSDs of the simulations are shown in Table 5. The RMSDs in vacuum range from 0.416 to 0.689, and are significantly higher than those of the simulations with CG solvent. The reason for this obvious difference is that the initial structures of the simulations are the native structures of proteins which are maintained in a solvent environment. The maintenance of the native structure is determined largely by the balance of the interactions among different amino acid residues with each other and with the aqueous solution surrounding the protein, and the solvent influences the conformation by competing with intramolecular interactions. The bonded potential in this work is derived from a statistical analysis of a representative protein set, so the solvent effect is incorporated in an implicit way. However, the van der Waals interactions are determined via simulations in vacuum, so the solvent effect is not incorporated in the potential. Therefore, the RMSD values to the initial structures in the simulations will be larger when the CG solvents are absent.

## 3. Materials and Methods

### 3.1. The Coarse-Grained Protein Models

**Figure 9:**
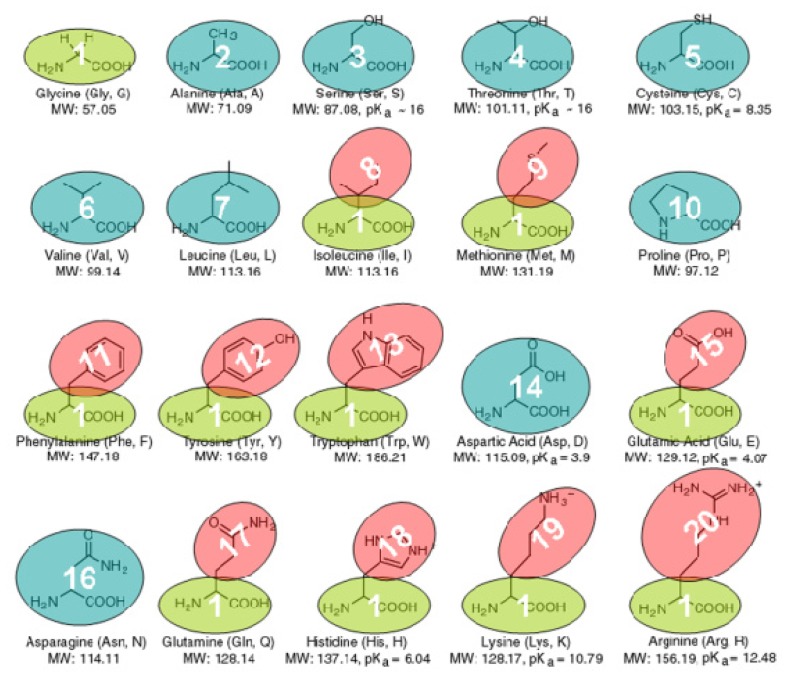
The coarse-grained models of 20 protein amino acids.

With our CG protein models, each amino acid is modeled by one or two beads according to their sizes. In total, 20 types of CG beads were designed for 20 amino acids as shown in Figure 9, which can be classified into two broad categories: backbone bead and side-chain bead. The backbone and side-chain beads can be denoted as *B_i_* (*i* = ALA, ASN, ASP, CYS, GLY, LEU, PRO, SER, THR, VAL) and *S_i_* (*i* = ARG, GLN, GLU, HIS, ILE, LYS, MET, PHE, TRP, TYR), respectively. Some amino acids are modeled only by one backbone bead due to their small side-chains, while others are modeled by one uniform backbone bead (Glycine bead *B_GLY_*) and one distinct side-chain bead. All the CG beads are idealized as a sphere, and center of the backbone bead is located at the alpha-carbon atom, while the center of the side-chain bead is located at the geometric center of all its heavy atoms.

### 3.2. The Coarse-Grained Force Field

**Figure 10:**
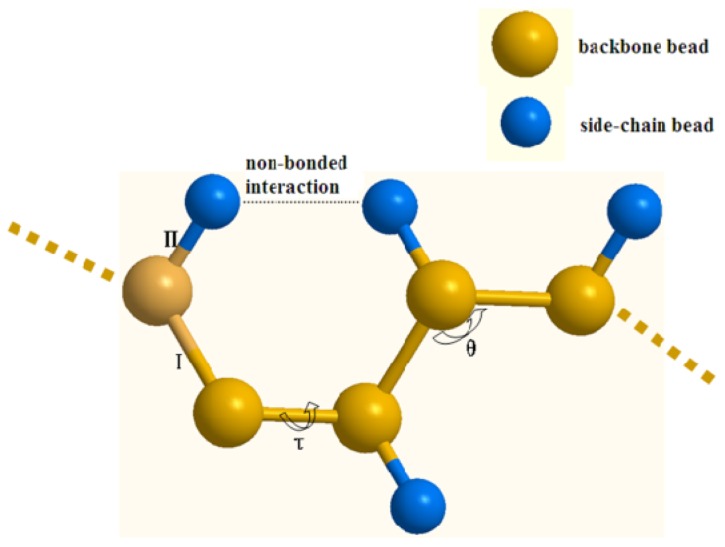
The coarse-grained protein model: I and II denote the backbone-backbone bead and backbone-side-chain bead bond stretching interaction respectively, θ denotes the virtual angle, and τ is the virtual dihedral angel.

With the above mentioned protein CG models, the structure and internal interactions of a protein can be simplified as shown in Figure 10. The CG force field can be formulated as Equation 1:

(1) $$U=\underline{U_{bond}+U_{angle}+U_{torsion}}+\underline{U_{vdw}+U_{elec}}$$

where *U_bond_*, *U_angle_* and *U_torsion_* are the stretching potential energy of a virtual bond, the potential energy of a virtual angle bending and the potential function of a dihedral angle about a rotating bond, respectively, which describe the bonded interactions between CG beads. *U_vdw_* and *U_elec_* describe the non-bonded interactions, which are the energy of van der Waals interactions and electrostatic interactions respectively.

### 3.3. The Bonded Potential and Parameterization

The virtual stretching interaction between two bonded CG beads can be described as a harmonic potential:

(2) $$U_{bond}=\sum \frac{1}{2}K_{bond}{\left(l-L_{bond}\right)}^{2}$$

where *K_bond_* and *L_bond_* are the force constant and the equilibrium stretching length of a bond, respectively, which will be determined by fitting the energy distribution of the virtual bond. Due to the coarse-graining, *U_angle_* and *U_torsion_* curves become more complex and irregular when compared with those of AA force field, and they are described with Gaussian distribution function:

(3) $$U_{angle/torsion}=\sum_{i=1}^{N}a_{i}\;\text{exp}[-{\left(\frac{x-b_{i}}{c_{i}}\right)}^{2}]$$

where *N*, *a_i_*, *b_i_* and *c_i_* are Gaussian parameters need to be determined in the parameterization process.

For correctly parameterize the bonded potential, we adopted a reduced and non-redundant set of protein structures used for fold recognition [39]. This set includes about 3600 structures chosen from the Protein Database Bank (PDB) [40], and the Root Mean Square Deviation (RMSD) of each structure is at least 6 Å to the rest of the structures in the set to avoid structure redundancy. Statistical analyses were performed against this protein set, and the resulting probability distributions were used to calculate Potential of Mean Force (PMF) via Boltzmann conversion method [13,41,42]:

(4) $$U_{i}=-k_{B}T\;\text{ln}(P_{i})$$

where *k_B_* is the Boltzmann constant, *T* is the temperature, and *P_i_= n_i_*/*n_ref_* is the probability of a property at value *i*, in which the reference number *n_ref_* is the total number of the investigated internal coordinate obtained from the statistics of the above mentioned protein set.

### 3.4. The Non-Bonded Potential and Parameterization

Modeling the non-bonded potential is a key problem of constructing CG-MD force filed. As in a classical AA force field, we assume that the non-bonded interaction can be subdivided into two categories, *i.e.*, van der Waals interaction and electrostatic interaction. They can be formulated as sums of pairwise potential energy:

(5) $$U_{vdw}=\sum_{i<j}4{ɛ}_{ij}\left(\frac{c_{ij}^{12}}{r_{ij}^{12}}-\frac{c_{ij}^{6}}{r_{ij}^{6}}\right)$$

(6) $$U_{elec}=\sum_{i<j}\frac{Q_{i}Q_{j}}{4\pi {ɛ}_{0}{ɛ}_{r}r_{ij}}$$

where *c_ij_* is the van der Waals interaction parameter, *r_ij_* is the distance between CG beads *i* and *j*, and *Q_i_* and *Q_j_* are the charges of *i* and *j*. The strength of the van der Waals interaction is determined by the value of well depth *ɛ_ij_* which depends on the types of the interacting CG beads and can be determined in the force field parameterization process for all the 20 types of CG beads. In the proposed force field, the electrostatic interaction is taken into account through distributed point charges, and four CG beads are treated as charged: backbone bead *B_ASP_* and side-chain bead *S_GLU_* are one unit negatively charged, and side-chain beads *S_ARG_* and *S_LYS_* are one unit positively charged. The electrostatic interaction between charged beads is calculated via Equation 6 with the relative dielectric constant *ɛ_r_* = 1.

In the coarse-graining, a group of atoms are treated as a single bead, and the relative positions of these atoms are fixed, but in reality, their relative positions vary in all the time. PMF is defined as the potential that gives an average force over all the configurations of a given system, and is used here to characterize the non-bonded interactions between CG beads. Umbrella Sampling (US) method [43] based on AA-MD was applied on the AA molecules of 20 CG beads to get the van der Waals well depth parameter when one molecule interacts with itself, and Lorentz-Berthelot mixing rules were applied for getting the interaction parameters between different CG beads:

(7) $$\begin{array}{l} c_{ij}^{6}={\left(c_{ii}^{6}c_{jj}^{6}\right)}^{1/2}\\ c_{ij}^{12}={\left(c_{ii}^{12}c_{jj}^{12}\right)}^{1/2} \end{array}$$

To perform the US simulations, backbone beads are simulated with corresponding AA amino acids, and side-chain beads are replaced by analogous compounds, as listed in Table 6. The simulations were performed with GROMACS 4.0.5 package, using fully flexible molecules in vacuum and the canonical NVT ensemble, and with vanishing charge in order to capture the purely non-electrostatic interaction. The GROMOS87 force filed was applied to the molecules, and the temperature is kept at 300 K by coupling to a Berendsen thermostat. Two identical AA molecules of a CG bead were placed together and equilibrated for 2000 ps, then two molecules were pulled from their equilibrium position to 15 angstroms along a reaction coordinate via umbrella pulling with a constant pulling rate 0.001 nm ps^−1^. The snapshots were saved every 1 ps, and the pulling distance was divided into subspaces every 0.5 angstrom. At last, US simulation was applied in every subspace for 10 ns, and the Weighted Histogram Analysis Method (WHAM) [44] was applied to accurately integrate the PMF of the non-bonded interaction between two homologue AA molecules.

### 3.5. Coarse-Grained Water Model and Parameterization

In protein simulation, quite often most of the computational cost is spent on calculating water-water intermolecular interactions rather than solute-water or solute-solute interactions, so the water coarse-graining will remarkably improve the efficiency of MD simulation. An appropriate water model should be constructed in the CG protein-solvent system, and the CG water model should be consistent with the protein CG models both in volume and mass, so the interactions between protein and solvent can be accurately reproduced. For determining the appropriate coarse-graining methodology of water molecules, we took a simulation of pure water with GROMACS 4.0.5 package with TIP3P water model, using GROMOS87 force field and the canonical NPT ensemble. The system was coupled to a temperature bath at 300 K and a barostat at 1 bar pressure, and was simulated for 1 ns. Statistical analysis with the simulation results showed that the average volume of five water molecules is about 140 Å^3^ and the mass is 90 amu, which is consistent with the average volume 120 Å^3^ and average mass 95 amu of the proposed CG protein beads. Therefore, the CG solvent model composed of five water molecules is adopted. The CG water bead is treated as neutral according to its total charge, so the interactions between CG water beads and other CG beads are mainly through van der Waals force. In order to determine the parameters of the van der Waals function for the CG water bead, every five nearest water molecules were clustered into a group with K-means algorithm, and the nearest distances between a group and the adjacent groups were calculated. According to the distribution probability, 0.51 nm is adopted for the parameter *c_ij_* in Equation 5. Using identical settings with the previous AA-MD water simulation, CG water system was simulated with different *ɛ_ij_*. For determining the best well depth parameter, the bulk density of the CG water system as a function of time was calculated and compared with the density variation of AA-MD. According to the comparison, *ɛ_ij_* = 6 kJ mol^−1^ is the best value for reproducing the bulk phase density of water, and is adopted in our CG force field.

## 4. Conclusions

In this work, 20 CG beads for protein were constructed according to the characters of 20 amino acids, and a residue is composed of only one or two CG beads. Correspondingly, with the K-means method, a five-water coarse-grained solvent model was adopted to suit the CG protein model. A force field was developed for the CG protein and solvent model. For all the bonded interactions in protein, CG beads are divided into two types. All the combinations with these two types of the bonded interactions were analyzed on a known protein structure subset, and the resulting energy distributions were fitted to various potential functions to formulate the bonded interactions in CG-MD. The umbrella sampling method was used on the AA molecules of the CG beads to get the PMF of non-bonded interactions between CG beads, and the PMF was fitted to a Lennard-Jones function potential to describe the non-bonded interactions in CG-MD.

The CG model and force field were tested on eight small to medium size proteins. With the results analysis of the simulations, without any extra information of the simulated protein structure, the skeleton structure of the protein can be maintained during a long time equilibrium dynamics simulation with the proposed coarse-graining methodology. Comparison of the efficiency shows that the proposed CG-MD can make a 75~100 computing speedup relative to AA-MD, which is also higher than the popular MARTINI model. Meanwhile, the native structure of the proteins can be well preserved during the simulations. In addition, RMSFs of Cα atoms during the simulation show our CG-MD method can reasonably sampling the conformational fluctuations within a protein from a global perspective. However, the simulation results also indicate that the fluctuation of loop structures with a low curvature in protein may be overestimated in some proteins compared with experimental values, and the situation is converse when the loop structures have a high curvature because the simplification in the CG-MD. Further work is needed to investigate and carefully treat the loop structures in protein coarse-graining methodology.

## Supplementary Information



## Figures and Tables

**Table 1:** The equilibrium length between ten side-chain beads and their backbone beads.

Bond	Length (nm)	Bond	Length (nm)
*B*–*S_ARG_*	0.406	*B*–*S_LYS_*	0.344
*B*–*S_GLN_*	0.301	*B*–*S_MET_*	0.287
*B*–*S_GLU_*	0.295	*B*–*S_PHE_*	0.333
*B*–*S_HIS_*	0.307	*B*–*S_TRP_*	0.381
*B*–*S_ILE_*	0.226	*B*–*S_TYR_*	0.371

**Table 2:** The finite distance *c_ij_* when the van der Waals potential of two interacting beads is equal to zero.

Interacting beads	Distance *c_ij_* (nm)	Interacting beads	Distance *c_ij_* (nm)
*B_ALA_*–*B_ALA_*	0.50	*S_ARG_*–*S_ARG_*	0.60
*B_ASN_*–*B_ASN_*	0.60	*S_GLN_*–*S_GLN_*	0.45
*B_ASP_*–*B_ASP_*	0.55	*S_GLU_*–*S_GLU_*	0.45
*B_CYS_*–*B_CYS_*	0.50	*S_HIS_*–*S_HIS_*	0.45
*B_GLY_*–*B_GLY_*	0.40	*S_ILE_*–*S_ILE_*	0.50
*B_LEU_*–*B_LEU_*	0.55	*S_LYS_*–*S_LYS_*	0.45
*B_PRO_*–*B_PRO_*	0.65	*S_MET_*–*S_MET_*	0.45
*B_SER_*–*B_SER_*	0.50	*S_PHE_*–*S_PHE_*	0.45
*B_THR_*–*B_THR_*	0.50	*S_TRP_*–*S_TRP_*	0.65
*B_VAL_*–*B_VAL_*	0.50	*S_TYR_*–*S_TYR_*	0.55

**Table 3:** The simulation information of eight protein systems.

System	PDB ID	Number of residues	Number of CG waters	Number of CG beads
Barstar	1BTA	89	939	1069
CheY	1CYE	129	1196	1375
Ubiquitin	1D3Z	76	1013	1124
FKBP12	1FKS	107	1264	1417
Barnase	1FW7	110	1157	1312
RNase H	1RCH	155	1982	2207
RNase A	2AAS	124	1126	1296
protein G	3GB1	56	887	963

**Table 4:** Resulting root mean square deviations from experimental structures of eight proteins during coarse-grained simulations compared with all-atom simulations (standard deviations are given in parentheses).

PDB	CG-MD			AA-MD *		
	
	Simulation length (ns)	Avg. Ca RMSD (nm)	Final Ca RMSD (nm)	Simulation length (ns)	Avg. Ca RMSD (nm)	Final Ca RMSD (nm)
1bta	1000	0.393(0.010)	0.396	142.9	0.134(0.016)	0.121
1cye	1000	0.389(0.036)	0.422	124.7	0.143(0.020)	0.170
1d3z	1000	0.394(0.020)	0.395	22.0	0.141(0.021)	0.128
1fks	1000	0.379(0.021)	0.415	143.5	0.358(0.074)	0.477
1fw7	1000	0.391(0.033)	0.408	148.0	0.171(0.015)	0.167
1rch	1000	0.415(0.025)	0.431	121.5	0.278(0.017)	0.289
2aas	1000	0.364(0.034)	0.400	148.3	0.249(0.043)	0.321
3gb1	1000	0.316(0.015)	0.323	50.0	0.106(0.020)	0.143

* The values of AA-MD are from reference 37.

**Table 5:** The efficiency of 10 ns simulations of eight proteins with three different simulation methodologies.

PDB	The proposed CG-MD			MARTINI		AA-MD	
		
	Simulation time (s)	Avg. Ca RMSD (nm)	Avg. Ca RMSD in vacuum (nm)	Simulation time (s)	Avg. Ca RMSD (nm)	Simulation time (s)	Avg. Ca RMSD (nm)
1bta	3501	0.210	0.637	4002	0.341	313062	0.148
1cye	4309	0.292	0.440	4972	0.503	398432	0.148
1d3z	4032	0.283	0.416	4261	0.426	334203	0.185
1fks	4484	0.324	0.505	5242	0.378	436792	0.220
1fw7	4391	0.247	0.574	4902	0.400	388712	0.171
1rch	7330	0.337	0.681	7845	0.357	650507	0.234
2aas	4424	0.284	0.689	4801	0.421	387623	0.259
3gb1	3432	0.275	0.501	3721	0.339	274854	0.128

**Table 6:** The corresponding analogous compounds of side-chain beads.

Side-chain bead	Analogous compound	Side-chain bead	Analogous compound
*S_ARG_*	*n*-propylguanidine	*S_LYS_*	*n*-butylamine
*S_GLN_*	propionamide	*S_MET_*	methyl propyl sulfide
*S_GLU_*	propionic acid	*S_PHE_*	toluene
*S_HIS_*	4-methylimidazole	*S_TRP_*	3-methylindole
*S_ILE_*	*n*-butane	*S_TYR_*	p-cresol
