# Supplementary material for: A Generic Force Field for Protein Coarse-Grained Molecular Dynamics Simulation
Source: Int J Mol Sci. 2012 Nov 8;13(11):14451–69. doi: 10.3390/ijms131114451 (PMC3509591; doi:10.3390/ijms131114451)
Supplement: Supplementary file 1 [file ijms-13-14451-s001.pdf]

Supplementary Information

Table S1. The Gaussian parameters for the bending and torsion energy potentials. B denotes the backbone bead, and S_i denotes the side-chain bead.

	B–B–B	B–B–S_i	S_i–B–B	B–B–B–B	B–B–B–S_i	S_i–B–B–B	S_i–B–B–S_j
a_1	3.07×10^2	6.56×10^0	0.00×10^0	4.79×10^1	6.36×10^1	5.76×10^1	1.95×10^1
b_1	-1.50×10^0	4.43×10^0	0.00×10^0	-2.74×10^1	2.07×10^2	-8.28×10^1	-5.57×10^1
c_1	5.04×10^1	6.34×10^0	0.00×10^0	6.37×10^1	2.24×10^2	1.15×10^2	5.77×10^1
a_2	-8.43×10^2	1.46×10^1	2.46×10^2	1.64×10^1	6.20×10^1	5.66×10^1	1.80×10^1
b_2	5.79×10^1	1.30×10^2	-2.33×10^1	2.21×10^1	-2.01×10^2	1.18×10^2	1.40×10^2
c_2	2.58×10^1	1.23×10^1	7.63×10^1	2.10×10^1	9.96×10^1	7.97×10^1	5.88×10^1
a_3	2.48×10^1	3.17×10^0	8.15×10^0	3.93×10^1	2.31×10^1	1.55×10^0	4.05×10^1
b_3	1.04×10^2	3.58×10^1	5.64×10^1	-1.55×10^2	-3.36×10^1	3.08×10^1	-1.95×10^2
c_3	1.53×10^1	7.85×10^0	5.73×10^0	1.30×10^2	5.52×10^1	2.09×10^1	7.28×10^2
a_4	-2.16×10^2	2.25×10^2	4.38×10^2	5.37×10^1	0.00×10^0	0.00×10^0	0.00×10^0
b_4	5.65×10^1	-1.69×10^1	3.35×10^2	1.42×10^2	0.00×10^0	0.00×10^0	0.00×10^0
c_4	1.80×10^1	7.75×10^1	1.14×10^2	6.81×10^1	0.00×10^0	0.00×10^0	0.00×10^0
a_5	1.98×10^2	7.36×10^{15}	1.42×10^1	2.35×10^1	0.00×10^0	0.00×10^0	0.00×10^0
b_5	2.25×10^2	1.70×10^3	1.17×10^2	8.20×10^1	0.00×10^0	0.00×10^0	0.00×10^0
c_5	6.72×10^1	2.69×10^2	1.84×10^1	2.42×10^1	0.00×10^0	0.00×10^0	0.00×10^0
a_6	1.11×10^3	0.00×10^0	0.00×10^0	0.00×10^0	0.00×10^0	0.00×10^0	0.00×10^0
b_6	5.77×10^1	0.00×10^0	0.00×10^0	0.00×10^0	0.00×10^0	0.00×10^0	0.00×10^0
c_6	2.40×10^1	0.00×10^0	0.00×10^0	0.00×10^0	0.00×10^0	0.00×10^0	0.00×10^0

© 2012 by the authors; licensee MDPI, Basel, Switzerland. This article is an open access article distributed under the terms and conditions of the Creative Commons Attribution license (http://creativecommons.org/licenses/by/3.0/).
